# Uncovering the *PML::RARA* Fusion in Cytogenetically Cryptic and FISH-Negative Acute Promyelocytic Leukemia—A Case Report and Comprehensive Literature Review

**DOI:** 10.3390/genes16101159

**Published:** 2025-09-29

**Authors:** Busra N. Delikkaya, Jaime Eberle-Singh, Arianna B. Morton, Jerald Z. Gong, Jinglan Liu

**Affiliations:** 1 Hematopathology, Department of Pathology and Genomic Medicine, Thomas Jefferson University, Philadelphia, PA 19107, USA; busra.delikkaya@jefferson.edu (B.N.D.); jaime.eberle-singh@jefferson.edu (J.E.-S.); arianna.morton@jefferson.edu (A.B.M.); jerald.gong@jefferson.edu (J.Z.G.); 2 Clinical Cytogenomics Laboratory, Department of Pathology and Genomic Medicine, Thomas Jefferson University Hospital, Philadelphia, PA 19107, USA

**Keywords:** acute promyelocytic leukemia, APL, *PML::RARA* fusion, cytogenetics, FISH, RT-PCR, genomics, leukemogenesis

## Abstract

The *PML::RARA* fusion resulting from t(15;17) is the genetic hallmark of acute promyelocytic leukemia (APL), typically detected by cytogenetics and/or fluorescence in situ hybridization (FISH) studies. Rarely, APL patients present with normal cytogenetics and FISH findings, complicating diagnosis and delaying life-saving therapy. We report a 23-year-old male with clinical, morphologic and immunophenotypic features consistent with APL but negative for FISH studies. Despite prompt initiation of all-trans retinoic acid (ATRA) based on clinical suspicion, the patient succumbed to intracranial hemorrhage. Quantitative reverse transcriptase PCR (qRT-PCR) confirmed a long isoform *PML::RARA* fusion. A review of 34 published cytogenetics- and FISH-negative cases since 1995 demonstrates that RT-PCR-based methods reliably detect cryptic fusions. While advanced genomic approaches may identify these fusions at higher resolution, their accessibility, complexity, cost, and turnaround time often limit diagnostic utility in the urgent setting of APL. Given the extreme rarity of this subset, cytogenetics and FISH remain the standard frontline tests; however, these cases underscore the critical need to incorporate molecular testing into routine workflows. Early recognition and timely therapy are essential to reducing mortality in cryptic APL, and these cases also provide insight into mechanisms of atypical leukemia biology.

## 1. Introduction

Acute promyelocytic leukemia (APL), with approximately 800 new cases annually, accounts for approximately 10% of adult myeloid leukemia in the United States [1]. Morphologically, it is characterized by immature hypergranular promyelocytes with abundant cytoplasm, irregular nuclei, fine azurophilic granules, and Auer rods in bone marrow and/or peripheral blood. Immunophenotyping typically shows expression of CD13, CD33, CD117, and MPO, with absent or low expression of CD34, HLA-DR, and markers of granulocytic differentiation [2,3].

The cytogenetic hallmark of APL is the reciprocal translocation t(15;17)(q22;q21) involving the *PML* gene on 15q24 and the *RARA* gene on 17q21. This fusion blocks myeloid maturation at the promyelocytic stage but generally confers a favorable prognosis due to sensitivity to all-trans retinoic acid (ATRA) and arsenic trioxide (ATO) [2,3,4]. Approximately 90% of patients carry the balanced translocation, while 6% have complex rearrangements and 0.7% have cryptic translocations; and all result in the oncogenic *PML::RARA* fusion, typically detectable by conventional karyotyping or fluorescence in situ hybridization (FISH) using a *PML/RARA* dual-fusion probe. The remaining 1~2% of cases involve non-*PML* fusion partners for *RARA*, which can be identified by karyotyping or by FISH with a *RARA* break-apart probe [5].

While karyotyping and FISH remain standard-of-care diagnostic approaches, RT-PCR provides greater resolution by defining fusion isoforms and is primarily applied to monitor minimal residual disease. Due to different breakpoints in the *PML* and *RARA* genes, three major chimeric isoforms (bcr1, bcr2, and bcr3) and more than 30 atypical isoforms of the *PML::RARA* fusion have been identified. With the exception of a few non-*PML* fusions (e.g., *ZBTB16/PLZF::RARA*), most patients respond well to ATRA- and ATO-based therapy, underscoring the importance of timely and accurate diagnosis [3,4]. Exceptionally rare cases of APL with classic clinical and morphological features but negative results by cytogenetics and FISH have been reported. In these patients, *PML::RARA* fusion transcripts can still be identified by RT-PCR, and excellent outcomes are achieved when treatment is initiated promptly [6,7].

Herein, we describe a new APL case with typical morphology and immunophenotype but negative cytogenetics and FISH results, in which a long *PML::RARA* transcript was identified by RT-PCR. We also review the clinicopathologic features of 34 similar cases reported since 1995 and discuss alternative molecular and genomic approaches that may aid diagnosis. Of note, APL cases with cytogenetic abnormalities involving chromosome 15 or 17, or with abnormal FISH signals, even if atypical, were excluded. Cases with *RARB, RARG*, or some *MLL* and *NPM1* rearrangements which can clinically and morphologically mimic typical APL with *RARA* rearrangements, are not addressed in this manuscript, as they have been thoroughly reviewed elsewhere [3,4]. Additionally, cases with incidental t(15;17) translocations lacking clinical symptoms or molecular evidence of gene fusions were also excluded.

## 2. Materials and Methods

### 2.1. Clinical and Pathological Examination

Clinical data, including history, presentation, laboratory findings, management, and disease course, were obtained from chart review. Peripheral blood smears were prepared and examined using the Wright-Giemsa method.

### 2.2. Flow Cytometry

Expression of cell surface and intracellular markers was assessed by flow cytometric immunophenotyping using the FACSLyric cytometer (Becton Dickinson, San Jose, CA, USA). A six-color panel including FITC, PE, PerCP-Cy5.5, PE-Cy7, APC, and APC-H7-conjugated antibodies was employed to evaluate lineage-specific antigens. Intracellular staining was performed using the Fix & Perm Cell Permeabilization Kit (Caltag Medsystems Ltd., Buckingham, UK), following surface staining. Data were analyzed using FACSLyric Suite and Beckman Coulter Kaluza analysis software version 1.2 with CD45 versus side scatter gating.

### 2.3. Conventional Cytogenetic and Interphase Fluorescence In Situ Hybridization (FISH) Studies

Standard direct harvest and 24 h unstimulated cell culture were conducted with peripheral blood cells. Interphase FISH was carried out using dual-color dual-fusion (DCDF) *PML/RARA* translocation and *RARA* break-apart (BAP) probe sets (CytoTest Inc., Rockville, MD, USA). A one-hour STAT FISH was performed according to the manufacturer’s instruction. Karyotyping and FISH images capture and analysis were conducted with the ASI system (Applied Spectrum Imaging, Carlsbad, CA, USA). Findings were analyzed and interpreted according to the International System for Human Cytogenetic Nomenclature (ISCN) 2020.

### 2.4. Literature Review

A PubMed search was performed using the keywords “acute promyelocytic leukemia,” “atypical,” “cryptic,” “cytogenetic negative,” and “FISH negative.” Articles published in both English and non-English were considered. No specific publication date range was applied to ensure comprehensive coverage of all available cases. The literature search was conducted independently by two researchers, and the senior author assessed all cases and finalized the list for inclusion. Reviews, case reports, and original research articles were all included. Clinical, cytogenetic, or molecular features of selected cases with atypical or cryptic acute promyelocytic leukemia were carefully reviewed. Based on the available data, only cases with typical normal DCDF *PML::RARA* FISH signals, typical normal *RARA* BAP signals, normal karyograms, or abnormal karyograms not involving chromosomes 15 and 17 were included.

## 3. Results

### 3.1. Case Study

**Figure 1:**
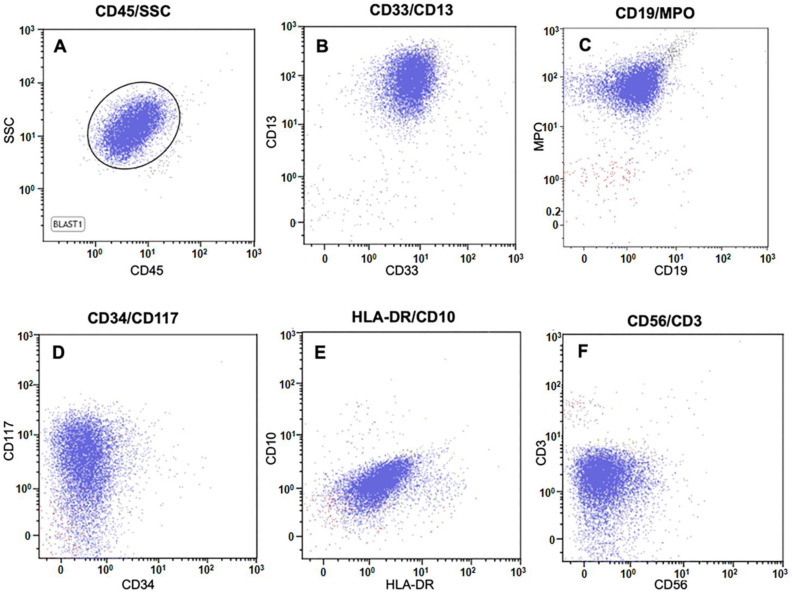
Flow cytometry analysis of peripheral blood identified blasts with high side scatter (**A**) that are positive for CD13, CD33 (**B**), MPO (**C**), and CD117 (**D**). Blasts are negative for CD34 (**D**), HLA-DR (**E**), and CD56 (**F**).

**Figure 2:**
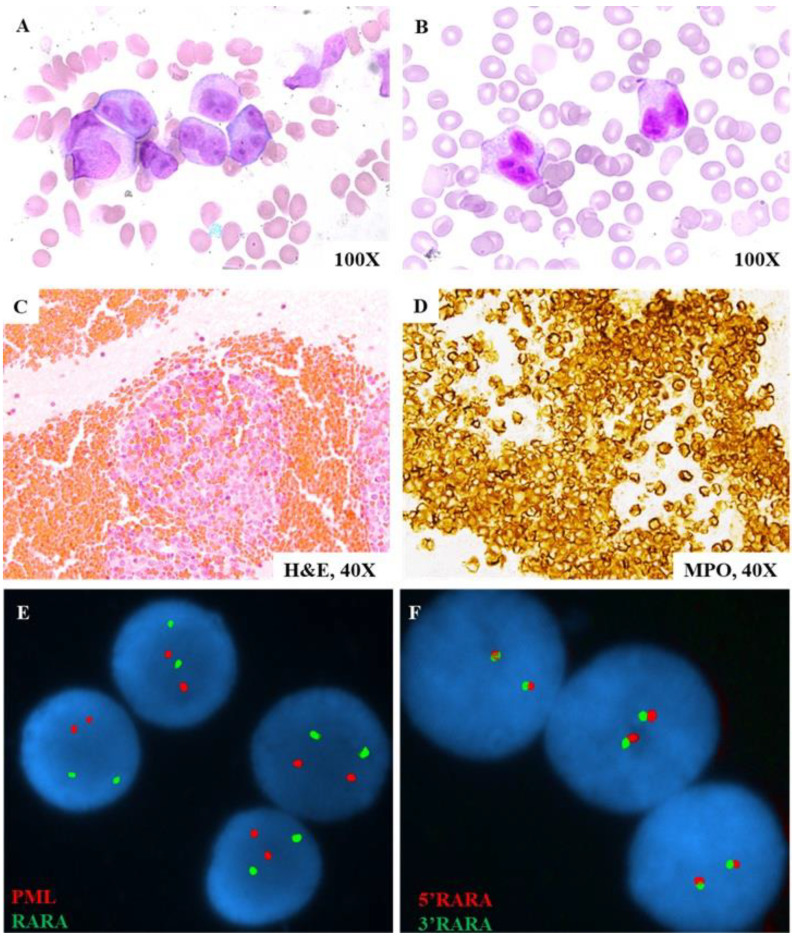
FISH and cellular morphological findings. (**A**,**B**) Peripheral blood smear demonstrating numerous promyelocytes with folded and bilobed nuclei with immature chromatin. (**C**,**D**) Right-sided temporectomy specimen demonstrating multiple brain parenchymal hemorrhages with dense clusters of MPO-positive leukemia cells. (**E**) Interphase FISH study using the DCDF *PML/RARA* probe set showing a normal pattern with two isolated read and green signals. (**F**) Interphase FISH study using the BAP *RARA* probe set showing a normal pattern with 2 fusion signals.

A 23-year-old male presented to the emergency department with left upper extremity weakness, occurring two days after undergoing a dental cleaning. Imaging revealed an intracranial hemorrhage, necessitating an urgent decompressive hemicraniectomy. Flow cytometry analysis demonstrated a cell population characterized by CD4−, CD10−, CD11b−, CD11c−, CD13+, CD14−, CD16−, CD33+, CD34−, CD38+, CD56−, CD64+(dim), CD117+, CD235a−, HLA-DR−, cCD3−, cCD22−, cCD79a−, MPO+, and TdT- expression (Figure 1). Peripheral blood smear examination identified abnormal promyelocytes (Figure 2A,B). These findings were consistent with acute promyelocytic leukemia (APL). However, STAT FISH analyses for *PML::RARA* fusion and *RARA* break-apart were negative (Figure 2E,F). Despite the absence of cytogenetic and FISH evidence, a diagnosis of APL was made based on the morphology and immunophenotypic profile. Treatment with all-trans retinoic acid (ATRA) was initiated immediately. Unfortunately, the patient’s post-operative course was complicated by severe coagulopathy, pancytopenia, and multi-organ failure, ultimately resulting in death seven days after admission. Histomorphological examination of the specimen from the right-sided temporectomy revealed multiple brain parenchymal hemorrhages with dense clusters of leukemia cells that stained positive for myeloperoxidase (MPO) (Figure 2C,D). Subsequent reverse transcriptase-polymerase chain reaction (RT-PCR) analysis, performed at an external commercial laboratory, was reported a week later, confirmed the presence of a *PML::RARA* long fusion transcript. Thus, the diagnosis of APL was established despite the negative findings from conventional cytogenetic studies.

### 3.2. Literature Review

Table 1 summarizes the clinical, histomorphological, and molecular genetic profiles of 34 APL patients lacking cytogenetic or FISH evidence of t(15;17). The median age was approximately 35~40 years (range 12~67), with a nearly equal male-to-female distribution. Most presented with bleeding, cytopenias, and laboratory evidence of disseminated intravascular coagulation (DIC) at the time of hospital admission. Morphologically, most cases showed classic hypergranular promyelocytes. Two cases exhibited microgranular variants, one showed a mixture of classic hypergranular promyelocytes with a high proportion of microgranular promyelocytes, and one was consistent with AML-FAB M5. Although t(15;17) was not detected by cytogenetics or FISH, all patients showed *PML::RARA* fusion transcripts by RT-PCR. The long (bcr1) isoform was most common (14 cases), followed by the short (bcr3) isoform (12 cases). The variant (bcr2) isoform was rare, observed in 2 patients. One patient had both bcr1 and bcr2 isoforms. The remaining five patients were positive for fusion transcripts without specification of the isoforms. Additional abnormalities, including trisomy 8, del(5q), del(9q), and i(17)(q10), were observed in a minority of cases but did not affect the molecular diagnosis. Treatment strategies evolved over time. Early cohorts were managed with ATRA combined with chemotherapy, whereas more recent patients received ATRA with arsenic trioxide (ATO), with or without chemotherapy. Overall, most patients achieved hematologic remission, although early deaths still occurred, predominantly due to hemorrhagic complications (intracranial or pulmonary) and, less frequently, severe infections. Early mortality, largely due to hemorrhagic complications, remained a significant cause of treatment failure. Long-term survival data were not available, but the modern ATRA + ATO-based regimens appear to be associated with superior remission durability.

**Table 1:** Clinical, Histomorphological, and Molecular Genetic Profiles of 34 Cytogenetics- and FISH-Negative APL Cases Reviewed in This Study ^¥^.

No.	Age/ Sex	WBC (10^9^/L)	Hb (g/dL)	Plt (10^9^/L)	Pro-myelocytes	Immuno-phenotype	Coagulation Tests	DIC at Dx	Reason for Presentation	Karyogram	FISH ^#^	RT-PCR *	Treatment	Survival (Months) /Terminal Event	CR	PMID
1	39/F	242.2	8.8	20	Yes ^1^	CD13+, CD33+, MPO+, HLA- DR+; CD34−	PT↑, aPTT↑	Yes	History of CML with persistent fever, malaise, and weight loss	46,XX,t(9;22)[20]	ND	S (bcr3)	IDA + Ara-C	No (5) /Bilateral mycotic pneumonia	Yes	7736444 [8] (Emilia, 1995)
2	25/F	2.9	8.3	9	Yes	CD33+; HLA-DR−	PT↑, FDP↑, fibrinogen↓, aPTT (nl)	Yes	Purpura and nasal bleeding	46,XX,del(9)(q22)[20]	ND	L (bcr1)	IDA + ATRA + Ara-C	No (<1) /ICH	No	10484977 [9] (Yamamoto, 1999)
3	N/A	N/A	N/A	N/A	Yes	N/A	N/A	N/A	N/A	45,XY,add(2)(q37),−7,add(9)(p22)/45,idem,add(10)(p14)	ND	L (bcr1)	N/A	N/A	N/A	10942371 [10] (Grimwade, 2000)
4	N/A	N/A	N/A	N/A	Yes	N/A	N/A	N/A	N/A	46,XY[20]	ND	L (bcr1)	N/A	N/A	N/A	10942371 [10] (Grimwade, 2000)
5	N/A	N/A	N/A	N/A	Yes	N/A	N/A	N/A	N/A	46,XX[20]	ND	Positive (unclear variant)	N/A	N/A	N/A	10942371 [10] (Grimwade, 2000)
6	N/A	N/A	N/A	N/A	Yes	N/A	N/A	N/A	N/A	46,XX[20]	ND	S (bcr3)	N/A	N/A	N/A	10942371 [10] (Grimwade, 2000)
7	N/A	N/A	N/A	N/A	Yes	N/A	N/A	N/A	N/A	46,XY[29]	ND	Positive (unclear variant)	N/A	N/A	N/A	10942371 [10] (Grimwadel, 2000)
8	N/A	N/A	N/A	N/A	Yes	N/A	N/A	N/A	N/A	46,XY[20]	ND	V (bcr2)	N/A	N/A	N/A	10942371 [10] (Grimwade, 2000)
9	48/F	14.93	8.8	36	Yes	CD13+, CD33+, MPO+, CD56+; HLA-DR−, CD34−	PT↑, D-dimer↑, fibrinogen↓, AT (nl), aPTT (nl)	No	Vaginal bleeding, history of uterine myoma	47,XY,+8[14]/46,XX[2]	ND	S (bcr3)	IDA + ATRA	Yes (>1)	Yes	16797070 [11] (Han, 2007)
10	14/M	N/A	N/A	N/A	Yes/hypo-cellular	Confirmed APL, WHO criteria	N/A	N/A	N/A	46,XY[20]	ND	S (bcr3)	IDA + ATRA	No (10) /GVHD	Yes	17943164 [12] (Kim, 2008)
11	63/F	N/A	N/A	N/A	Yes	Confirmed APL, WHO criteria	N/A	N/A	N/A	46,XX[20]	ND	S (bcr3)	IDA + Ara-C	No (6) /Pulmonary hemorrhage	Yes	17943164 [12] (Kim, 2008)
12	44/F	1.5	12.6	49	Yes	CD13+, CD33+, MPO+; HLA-DR−, CD34−	N/A	N/A	Immature cells in peripheral blood	46,XX,i(17)(q10)[12]/46,XX[8]	ND	L (bcr1)	IDA + ATRA + Ara-C	Yes (>1)	Yes	18294238 [13] (Huh J, 2008)
13	50/F	101.73	9.9	16	Yes	CD13+, CD33+, CD117+, CD7+, MPO+; HLA-DR−, CD34−	PT↑, FDP↑, fibrinogen↓, D-dimer↑, aPTT (nl), AT (nl)	No	Cough, fever, and general weakness for a month	47,XY,+8[19]/46,XX[1]	ND	S (bcr3)	IDA + ATRA	Yes (>5)	Yes	19893344 [14] (Kim, 2009)
14	39/F	16.9	7.6	174	Yes	CD13+, CD33+, MPO+; HLA-DR−, CD34−	fibrinogen↓, FDP↑, D-dimer↑, PT (nl), aPTT (nl)	No	Two weeks of fatigue, five days of high fever and irregular uterine bleeding	46,XX,7q[7]+/46,XX[8]	ND	L (bcr1)	ATRA + ATO	Yes (>2)	Yes ^2^	19162322 [15] (Wang, 2009)
15	26/F	0.6	7.7	155	Yes	CD13+, CD33+, CD117+; HLA-DR−, CD34−	PT↑, INR↑, aPTT (nl)	No	Fever, pallor and bleeding gums	del(5q)	ND	S (bcr 3)	IDA + ATRA	Yes (>8)	Yes	19224461 [16] (Choughule, 2009)
16	33/F	1.39	7.1	9.8	Yes	CD13+, CD33+, CD117+; HLA-DR−, CD34−	Normal	No	Fever and fatigue	Normal	ND	S (bcr 3)	ATO	Yes (>12)	Yes	19224461 [16] (Choughule, 2009)
17	46/M	63.8	6.6	146	Yes	CD13+, CD33+, MPO+; CD117−, HLA-DR−, CD34−	PT↑, aPTT↑, INR↑	No	Three weeks of fatigue and intermittent fever	del(19p13), del(12q24.1), del(5)	ND	L (bcr1)	IDA + ATRA	Yes (>8)	Yes	19224461 [16] (Choughule, 2009)
18	18/M	16.5	9.5	37	Yes	CD13+, CD33+, CD45+, CD117+; HLA-DR−, CD34−	N/A	No	Hematuria and hematochezia	46,XY[20]	ND	L (bcr1), V (bcr2)	ATRA	Yes (>1)	N/A	21156244 [6] (Kim, 2010)
19	46/M	1.7	10.6	18	Yes	CD13+, CD33+, MPO+; HLA-DR−, CD34−	PT↑, INR↑, D-dimer↑	No	Gengival bleeding	92,XXYY[13]/46,XY[7]	ND	S (bcr3)	IDA + ATRA	Yes (>14)	Yes	20417966 [17] (Soriani, 2010)
20	24/M	64.3	N/A	N/A	Microgranular variant	CD13+, CD33+, CD117+; CD45+, CD34+; HLA-DR−	N/A	Yes	Suboccipital headaches, nausea, photophobia, gingival bleeding, debilitating nuchal rigidity, and low-grade fevers	46,XY[20]	ND	Positive (unclear variant)	ATRA + ATO + Ara-C	Yes (>2)	Yes	22018276 [18] (Lewis, 2011)
21	23/M	3.94	12	36	Yes	CD13+, CD33+; CD34−	N/A	N/A	Ecchymosis and thrombocytopenia	47,XX,+8[19]/46,XY[1]	ND	L (bcr1)	ATRA + Chemo	n/a	Yes	22402611 [19] (Yang, 2012)
22	57/M	6.82	8.9	40	Yes	CD13+, CD33+, CD117+; HLA-DR−, CD34−	N/A	N/A	Oral ulcer, odynophagia, anemia, and thrombocytopenia	46,XY[20]	ND	L (bcr1)	ATRA + ATO + Ara-C	Yes (>5)	Yes	23370423 [20] (Gruver, 2013)
23	67/M	1.9	10.8	89	Yes	MPO+; CD34−, HLA-DR−	PTT↓, fibrinogen↓, INR↑, D-dimer↑	Yes	Easy bruising, fatigue, pancytopenia	46,XY[20]	ND	Positive (unclear variant)	ATRA	N/A	N/A	25580502 [21] (Rashidi, 2014)
24	17/M	9.9	n/a	n/a	Yes	CD13+, CD33+, CD117+; HLA-DR−, CD34−, CD11b−	N/A	Yes	gum bleeding, multiple ecchymoses, abdominal pain, and fever	46,XY[20]	ND	L (bcr1)	ATRA + ATO + Chemo	Yes (>24)	Yes	24561214 [22] (Blanco, 2014)
25	25/M	5.2	11.6	30	Yes	CD117+, CD64+, CD123+, CD13+, MPO+; CD34−, HLA-DR−	PT↑, INR↑, TT↑	N/A	Gum bleeding for over 20 days	46,XY[20]	ND	S (bcr3)	ATRA + ATO + IDA	Yes (>2)	Yes	27995890 [23] (Wang, 2016)
26	66/M	2.95	78	7	No ^3^	CD7+, CD13+, CD33+, CD34+, Cd38+, CD117+, CD38, HLA-DR+, MPO+	PT↑, fibrinogen↑, D-dimer↑, aPTT (nl)	N/A	Petechiae or bruises on the lower limbs that lasted for half a month with intermittent fever and coughing	46,XY[20]	ND	V (atypical) ^4^	IDA + ATRA + Ara-C	Yes (>7)	Yes	31959056 [24] (Zhang, 2020)
27	57/F	n/a	9.7	57	Microgranular variant	CD13+, CD33+, CD34+, CD117+, MPO+, CD2+; HLA-DR−, CD11b−,	fibrinogen↓ D-dimer↑, PT (nl), PTT (nl),	No	Bruising and gingival bleeding over a two-week period	46,XX[20]	ND	L (bcr1)	ATRA + ATO	Yes (>1)	Yes	31809670 [25] (Schultz, 2020)
28	17/M	48.4	8.3	14	Yes	CD13+, CD33+, CD38+, CD45+, CD64+, CD117+, HLA-DR+ (small subset), MPO +; CD34−	fibrinogen↓, D-dimer↑, PTT↑, TT (nl)	Yes	Unresponsive during a seizure after two-day history of nausea, blood-tinged vomiting, lethargy, and right-sided weakness	46,XY[20]	ND	Positive (unclear variant)	ATRA	N/A	Yes	32366568 [1] (Mai, 2020)
29	12/F	22.5	7.4	16	Yes	CD33+, CD34+, MPO+, CD117+, HLA-DR+; CD11b−	fibrinogen↓, PT↑, D-dimer (nl)	Yes	Multiple ecchymoses	46,XX[20]	ND	S (bcr3)	ATRA + Chemo	Yes (>26)	Yes	32909480 [26] (Avgerinou, 2020)
30	56/F	0.7	8	96	Yes	CD13+, CD33+, CD117+, CD34+, MPO+, CD56+; HLA DR−	N/A	No	Generalized pruritis for 1 month, fever and headache for 1 week	46,XX[20]	ND	L (bcr1)	ATRA + DNR + Ara-C	No (<1) /Refractory septic shock with encephalopathy	No	33851647 [27] (Arumugam, 2021)
31	54/M	1.6	9.4	69	Yes	MPO+, CD117+; CD34−, HLA-DR−	N/A	N/A	Left leg swelling and a left femoral vein thrombosis, syncopal episode	46,XY[20]	ND	L (bcr1)	ATRA + ATO	N/A	Yes	35572917 [28] (Karlin, 2022)
32	27/M	4.67	15	144	Yes	CD13+, CD33+, MPO+; CD34−, HLA-DR−	PT↑, D-dimer↓, aPTT (nl), fibrinogen (nl),	no	Imaging findings of marrow heterogeneity in T12 during L4–L5 disk herniation work-up	46,XY[20]	ND	L (bcr1)	ATRA + ATO	Yes (n/a)	Yes	37685882 [29] (Mohebnasab, 2023)
33	32/F	1.57	8.1	68	Yes	CD2+, CD13+, CD33+, CD117+, MPO+	PT↑, fibrinogen↓, D-dimer↑, aPTT (nl),	Yes	Several weeks of fatigue and easy bruising	46,XX[20]	ND	S (bcr3)	ATRA + ATO	Yes (n/a)	Yes	37685882 [29] (Mohebnasab, 2023)
34 ^&^	23/M	80.1	4.8	22	Yes	CD13+, CD33+, CD38+, CD64+, CD117+, MPO+; HLA-DR−, D34−,	PT↑, INR↑, PTT (nl)	N/A	Intracranial hemorrhage, 2 weeks post dental cleaning for pain	N/A	ND	L (bcr1)	ATRA	No (<1) /Acute respiratory failure secondary to ICH	No	N/A

Abbreviations: Dx: diagnosis; CR: complete remission; nl: normal; ND: not detected; N/A: not available; ATRA: all-trans retinoic acid; ATO: arsenic trioxide; IDA: Idarubicin; Ara-C: Cytarabine; AT: antithrombin; PT: prothrombin time; aPTT: activated partial thromboplastin time; PTT: partial thromboplastin time; FDP: fibrin degradation products; INR: international normalized ratio; GVHD: graft-versus-host disease; ICH: Intracranial hemorrhage; unk: unknown. ^1^ Mixture of classic hypergranular promyelocytes with an unusually high proportion of microgranular promyelocytes. ^2^ Hematologic complete remission without molecular remission. ^3^ Bone marrow cells displayed irregular nuclear shapes and misty nucleoli, weakly positive for POX cytochemical staining, consistent with AML-FAB M5. ^4^ Novel cryptic atypical V (bcr2) transcript. ^¥^ Two cases with normal karyograms and negative FISH results in the initial study [30,31] were excluded, as abnormal FISH findings were identified retrospectively. ^#^ Includes testing with dual-color, dual-fusion *PML/RARA* translocation probe set and/or break-apart *RARA* probe. * Includes RT-PCR, qRT-PCR, and multiplex nested RT-PCR, with or without subsequent cDNA sequencing. ^&^ The current case.

## 4. Discussion

APL is a rare and aggressive AML subtype defined by the *PML::RARA* fusion. Conventional karyotyping and FISH are first-line diagnostic tests, while RT-PCR-based assays confirm transcript isoforms and enable evaluation of minimal residual disease (MRD) during clinical follow-ups. Rarely, patients with typical clinical and histomorphological features and a favorable response to ATRA+ATO therapy lack detectable *PML::RARA* fusion by these assays [6,7]. We systematically reviewed 34 reported cases of cytogenetics- and FISH-negative APL published since 1995, including both English- and non-English-language reports. To the best of our knowledge, this constitutes the most comprehensive review of this rare entity to date.

Clinically, these patients closely resemble those with classic t(15;17) translocations, showing comparable hematologic profiles, immunophenotypes, and coagulopathy features (Table 1). Mechanistically, submicroscopic insertions are believed to cause the fusion in most cases [9,10,30,31,32]. These insertions often involve multiple breakpoints and are mediated by microhomology-mediated break-induced replication (MMBIR) or fork stalling and template switching (FoSTeS), resulting in the formation of the *PML::RARA* fusion. This represents a distinct genomic event from the conventional reciprocal t(15;17), which arises via non-allelic homologous recombination (NAHR) or breakage at palindromic AT-rich repeats (PATRRs) [33,34]. Together, these diverse mechanisms suggest that genes involved in DNA replication or repair may drive leukemogenesis even prior to fusion formation, underscoring the potentially distinct biology of cryptic APL.

Diagnostic approaches reported in the literature are summarized in Table 2, with a focus on diagnostic yield, turnaround time (TAT), potential pitfalls, and clinical applicability. Conventional karyotyping remains foundational, detecting the classic t(15;17) translocation and additional chromosomal abnormalities in over 96% of cases [5]. However, its sensitivity is reduced in atypical cases due to dependence on high-quality metaphase spreads and inability to resolve cryptic rearrangements. Nevertheless, its genome-wide scope allows detection of co-existing abnormalities with prognostic or therapeutic significance. FISH assays offer rapid, high-sensitivity detection of typical and atypical fusions (98~100% yield) when *PML/RARA* dual-color dual-fusion and *RARA* break-apart probes are used in parallel [3,32,35]. Their short TAT (~3 h) makes them valuable for urgent diagnostic confirmation, particularly in life-threatening coagulopathy. Limitations include hybridization failure, signal overlap, and inability to detect very small insertions, which may account for the rare subset of cytogenetics- and FISH-negative cases. Nonetheless, their low-to-moderate cost and widespread availability ensure their continued role as frontline diagnostic tools. RT-PCR-based molecular assays provide rapid, high-resolution detection of *PML::RARA* isoforms and are essential for MRD monitoring, with qRT-PCR considered the gold standard [18,26]. These assays are widely available and affordable, though rare atypical breakpoints may generate false negatives [36]. Sequencing of RT-PCR products allows precise characterization of fusion variants, albeit with longer TAT and higher RNA quality requirements. Advanced genomic technologies, including array comparative genomic hybridization (aCGH/tCGH), mate-pair sequencing (Mpseq), and optical genome mapping (OGM), enable detection of cryptic structural variants across broader genomic regions [30,37]. OGM, in particular, shows promise for resolving structural rearrangements and fusion breakpoints at a medium-level resolution [9]. However, these platforms require high-quality input material and specialized expertise, and challenges remain in detecting polyploidy, mosaicism, or rearrangements in heterochromatic or highly repetitive regions. TATs are longer (minimum 3~5 days), costs higher, and access restricted to specialized centers. At the highest resolution, rapid NGS panels, whole-genome and whole-exome sequencing (WGS/WES) provide targeted or comprehensive detection of fusion partners and profiling of co-existing aberrations [28,37]. Their application remains largely research-focused due to high cost, bioinformatics complexity, interpretation challenges, longer TAT, and the need for variant validation.

Collectively, these methods form a complementary, hierarchical diagnostic framework (Table 3). First-line testing with karyotyping, FISH, and RT-PCR is sufficient for most cases, offering high diagnostic yield, short TAT, and wide accessibility at relatively low cost. Advanced technologies such as OGM and WGS/WES are best reserved for ambiguous cases where conventional techniques fail, as well as for expanding our understanding of APL genomics. This tiered approach balances diagnostic precision with practical feasibility, while addressing the small but clinically significant subset of cytogenetics- and FISH-negative APL.

This review is limited by the small number of reported cases and incomplete documentation of clinical and demographic data, which precludes meaningful statistical analysis. The 34 cases identified across 25 manuscripts, including the present study (Table 1), lacked consistent information on ethnicity and racial background. Thirteen studies (52%) originated from groups in China, India, Japan, and Korea, and including our patient from India, 17 patients (50%) were presumably of Asian descent. Given the limited demographic, epidemiological, and socioeconomic data, no conclusions can be drawn about the role of genetic background or environmental factors in the pathogenesis of this ultra-rare APL subset. Furthermore, all studies were retrospective in design. Most were single case reports, with a few small case series, which limits the generalizability of the findings. Potential reporting bias and heterogeneity of the available data could not be confidently assessed. Supplementary Table S1 summarizes the limitations and potential bias for each case. These cases, however, point to a possible association between cryptic APL and ethnicity/race, emphasizing the need for more detailed reporting in future cases.

Despite the small cohort size, several important questions remain. First, it is unclear whether cryptic rearrangements detected by RT-PCR-based methods are balanced or unbalanced, as unbalanced fusions may acquire novel functions that influence phenotypic outcomes [38]. Second, the mutational spectrum of co-existing primary or secondary genetic alterations is poorly defined. Experimental studies in transgenic mice suggest that cooperating mutations are essential to drive full leukemic transformation [39]. In de novo APL, co-existing mutations, including point mutations in the *PML::RARA* fusion gene, occur in approximately ~70% of cases and also significantly contribute to relapse or therapy resistance [40,41]. These mutations are heterogeneous but may converge functionally to drive APL initiation and confer therapy resistance [23,24]. Third, the impact of such alterations on higher-order chromosome architecture, epigenetic regulation, and transcriptional networks remains largely unknown.

Clinically, APL patients lacking cytogenetic or FISH evidence face high risk of diagnostic delay and potentially fatal early hemorrhage [28]. Empiric initiation of ATRA upon clinical suspicion remains essential, even when first-line tests are negative. Emerging approaches, including OGM, long-read sequencing, and multi-omics technologies (epigenomics, transcriptomics, metabolomics), hold promise for illustrating cryptic mechanisms and revealing therapeutic targets. Despite major therapeutic advances, early mortality of APL remains ~15%. Studying this ultra-rare subset may not only improve outcomes in cryptic APL but also yield broader insights into APL biology and leukemogenesis.

## Figures and Tables

**Table 2:** Comparison of methods reported in the literature for evaluating cytogenetics- and FISH-negative APL cases. All performance metrics refer exclusively to APL patients.

Method	Application	Abnormalities Detected	APL Yield	STAT TAT	Causes of Error	Availability	Cost
Karyotyping	Standard of care	t(15;17) and all other chromosomal alterations	>96%	2 days	Poor metaphase quality, culture failure, submicroscopic cryptic translocations	Widely available	Low
FISH–PML/RARA DCDF	Standard of care; Diagnostic confirmation	Typical and atypical *PML::RARA* fusion	~98%	3 h	Hybridization failure, signal overlap, small insertions leading to absent or weak signals	Widely available	Low–Moderate
FISH–RARA BAP	Standard of care; Diagnostic confirmation	*RARA* rearrangements (*PML* and non-*PML* partners)	~100%	3 h	Hybridization failure, signal overlap, small insertions leading to absent or weak signals	Widely available	Low–Moderate
RT-PCR	Diagnostic confirmation	*PML::RARA* transcript isoforms	>95%	4 h	Sample contamination, primer errors due to novel breakpoints	Widely available	Low–Moderate
qRT-PCR	MRD monitoring	*PML::RARA* transcript isoforms	>95%	4 h	Sample contamination, primer errors due to novel breakpoints	Widely available	Low–Moderate
RT-PCR + cDNA sequencing	Diagnostic confirmation	*PML::RARA* transcript isoforms, fusion breakpoints	>95%	2 days	Sequencing errors, mis-priming, low-quality RNA	Moderate	Moderate
tCGH/cCGH/Mpseq	Advanced genomic assessment for rare cases (at low resolution)	*PML::RARA* transcript isoforms; additional non-diagnostic genomic aberrations	Unknown, very limited studies to date	3 days	Undetectable balanced rearrangements and unmapped sequences, oversight of low-level mosaicism, inaccurate CNV calls	Limited	Moderate
OGM	Advanced genomic assessment for rare cases (at medium resolution)	*PML::RARA* and non-*PML::RARA* transcript isoforms; fusion breakpoints; additional non-diagnostic genomic aberrations	Unknown, requires further validation for standard APL diagnostics	3~5 days	Poor recovery of HMW DNA, complex rearrangement miscalls, undetectable polyploidy, unresolved centromeric/p-arm rearrangements, missed low-level mosaicism	Limited	Moderate–high
WGS/WES	Advanced genomic assessment for rare cases (at high resolution)	*PML::RARA* and non-*PML::RARA* transcript isoforms; fusion breakpoints; additional non-diagnoatic genomic aberrations	Unknown, primarily used in research or complex cases rather than routine APL diagnosis	3~5 days	Sequencing artifacts, bioinformatics miscalls, over-sight of low-level mosaicism, variant validation challenges, interpretation challenges, conditional detection of structural rearrangements	Limited	High

**Abbreviations:** cCGH: chromosomal comparative genomic hybridization; tCGH: targeted Comparative Genomic Hybridization; MPseq: mate-pair sequencing; OGM: Optical genome mapping; HMW: high-molecular-weight; TAT: turn around time; WES: whole-exome sequencing; WGS: whole-genome sequencing.

**Table 3:** Stepwise approach to maximize diagnostic yield in cytogenetics- and FISH-negative APL.

Tier	Methods	Clinical Relevance
First line	FISH (DCDF and BAP), Karypotying, RT-PCR	High-yield, fast, easily accessible
Advanced	OGM, CMA, targeted sequencing, long read sequencing	Complex cases
High-resolution/ research-oriented	WGS/WES, other omics	High-complexity cases, with inconclusive results

## Data Availability

All supporting data for this study are included in this manuscript. Additional inquiries may be directed to the corresponding author.

## Supplementary Materials

The following supporting information can be downloaded at: https://www.mdpi.com/article/10.3390/genes16101159/s1, Table S1. Study-Specific Limitations and Potential Biases in Reported Cytogenetics- and FISH-Negative APL Cases.
