# Supplementary material for: Uncovering the PML::RARA Fusion in Cytogenetically Cryptic and FISH-Negative Acute Promyelocytic Leukemia—A Case Report and Comprehensive Literature Review
Source: Genes (Basel). 2025 Sep 29;16(10):1159. doi: 10.3390/genes16101159 (PMC12564908; doi:10.3390/genes16101159)
Supplement: Supplementary file 1 [file genes-16-01159-s001.zip › genes-3872364-supplementary.pdf]

Supplementary Table S1. Study-Specific Limitations and Potential Biases in Reported Cytogenetics- and FISH-Negative APL Cases

Year	Author	Sample Size	Key Findings	Study Type	Limitations	Potential Bias
1995	Emilia G., et al	1	t(9;22) by karyotype; *PML::RARA* by RT-PCR	Singel case report	Limited demographic information, incomplete clinical follow-up	Case selection bias
1999	Yamamoto JF., et al	1	*PML::RARA* by RT-PCR	Singel case report	Llimited demographic information, limited immunophenotypic panel, incomplete clinical follow-up,	Case selection bias
2000	Grimwade D., et al./European working party	6	*PML::RARA* by RT-PCR	Multicenter study	Variability in diagnostic assays and reporting, no demographic and clinical data, no follow-up,lack of additional genomic data	Case selection bias
2007	Han JY., et al.	1	Trisomy 8 by karyotype; *PML::RARA* by RT-PCR	Singel case report	Limited demographic information, incomplete clinical follow-up	Case selection bias
2008	Kim M., et al.	2	*PML::RARA* by nested RT-PCR	Small case series	No demographic information except age and sex, no specific clinical information except a diagnosis, incomplete clinical follow-up	Case selection bias
2008	Huh J., et al.	1	i(17)(q10) by karyotype; *PML::RARA* by RT-PCR	Singel case report	Limited demographic information, incomplete clinical follow-up	Case selection bias
2009	Kim KE., et al.	1	Trisomy 8 by karyotype; *PML::RARA* by RT-PCR	Singel case report	Non-English, limited demographic information, incomplete clinical follow-up	Case selection bias
2009	Wang Y., et al.	1	7q+ by karyotype;	Singel case report	Incomplete karyotyping, limited demographic	Case selection bias

			PML::RARA by RT-PCR		information, incomplete clinical follow-up	
2009	Choughule A., et al.	3	Various cytogenetic abnormalities by karyotype; *PML::RARA* by RT-PCR	Small case series	Variability in diagnostic assays and reporting; limited demographic information, incomplete clinical follow-up	Case selection bias
2010	Kim MJ., et al.	1	*PML::RARA* by RT-PCR	Singel case report	Incomplete clinical data and follow-up	Case selection bias
2010	Soriani S. et al.	1	Tetraploidy by karyotype; *PML::RARA* by RT-PCR	Singel case report	Incomplete clinical follow-up	Case selection bias
2011	Lewis C., et al,	1	*PML::RARA* by RT-PCR	Singel case report	Incomplete clinical data and follow-up	Case selection bias
2012	Yang JJ., et al.	1	Trisomy 8 by karyotype; *PML::RARA* by RT-PCR	Singel case report	Incomplete clinical data and follow-up	Case selection bias
2013	Gruver AM., et al.	1	*PML::RARA* by RT-PCR	Singel case report	Incomplete clinical data and follow-up	Case selection bias
2014	Rashidi A. et al.	1	*PML::RARA* by RT-PCR	Singel case report	Unclear variant, incomplete clinical follow-up	Case selection bias
2014	Blanco EM., et al.	1	*PML::RARA* by RT-PCR	Singel case report	Limited demographic information, incomplete clinical follow-up	Case selection bias
2016	Wang YF., et al.	1	*PML::RARA* by RT-PCR	Singel case report	Non-English, limited demographic information, incomplete clinical follow-up	Case selection bias
2020	Zhang Z., et al.	1	*PML::RARA* by RT-PCR	Singel case report	Limited demographic information, incomplete clinical follow-up	Case selection bias
2020	Schultz MJ.,m et al.	1	*PML::RARA* by RT-PCR	Singel case report	Limited demographic information, incomplete clinical follow-up	Case selection bias
2020	Mai B., et al.	1	*PML::RARA* by RT-PCR	Singel case report	Unclear variant, limited demographic information, incomplete clinical follow-up	Case selection bias

2020	Avgerinou G., et al.	1	*PML::RARA* by RT-PCR	Singel case report	Limited demographic information, incomplete clinical follow-up	Case selection bias
2021	Arumugam JR., et al.	1	*PML::RARA* by RT-PCR	Singel case report	Limited demographic information, incomplete clinical data and follow-up	Case selection bias
2022	Karlin K., et al.	1	*PML::RARA* by RT-PCR	Singel case report	Limited demographic information, incomplete clinical data and follow-up	Case selection bias
2023	Mohebnasab M., et al.	2	*PML::RARA* by RT-PCR	Small case series	Variability in diagnostic assays and reporting; limited demographic information, incomplete clinical follow-up	Case selection bias
2025	Current report	1	*PML::RARA* by RT-PCR	Singel case report	Limited demographic information, incomplete clinical follow-up	Case selection bias
